# Psychological burden, sexual satisfaction and erectile function in men whose partners experience recurrent pregnancy loss in China: a cross-sectional study

**DOI:** 10.1186/s12978-016-0188-y

**Published:** 2016-06-13

**Authors:** Yi-xiang Zhang, Xue-qi Zhang, Qing-rong Wang, Ye-qing Yuan, Jiang-gen Yang, Xiao-wei Zhang, Qing Li

**Affiliations:** Urology, The Second Clinical Medical College of Jinan University, Shenzhen People’s Hospital, Shenzhen, 518020 China; Urology, Peking University People’s Hospital, Beijing, 100044 China

**Keywords:** Psychological burden, Sexual satisfaction, Erectile function, Recurrent pregnancy loss

## Abstract

**Background:**

The aim of this study was to elucidate recurrent pregnancy loss (RPL)-associated psychosocial effects and sexual functions of Chinese men whose partners experience a history of RPL.

**Methods:**

Questionnaire data from a total of 236 men whose partners experience RPL(RPL group) and another 236 non-RPL male volunteers(control group) were analyzed. The self-administered questionnaires included anxiety and depression measures (SAS & SDS), the Index of Sexual Satisfaction (ISS) and the International Index of Erectile Function (IIEF-5) for evaluating psychological burden, sexual satisfaction and erectile function, respectively.

**Results:**

The mean age of the RPL group and control group was 29.8 ± 8.6 and 28.2 ± 7.3, respectively. The incidence of erectile dysfunction was significantly higher in the RPL group than in the control group (19.07 % vs. 7.63 %, *P* < 0.001). Anxiety and depression were also more prevalent in RPL group than in the control group (anxiety: 36.90 % vs. 19.08 %, *P* < 0.001; depression: 26.30 % vs. 7.63 %, *P* < 0.001). Furthermore, after adjusting for age in the RPL group, negative relationships were observed between the IIEF-5 score and anxiety and depression (*P* < 0.001), and a positive correlation was found between the ISS and anxiety and depression (*P* < 0.001). In addition, history of RPL, anxiety and depressive symptoms were significantly associated with a higher risk of ED.

**Conclusions:**

Psychological functioning, sexual satisfaction and erectile function are impaired in infertile men with RPL partners. These men should be targeted for psychological consultation.

## Background

Recurrent pregnancy loss (RPL), defined as two or more consecutive pregnancy losses before the 20th week of pregnancy, is a frequent obstetric complication [1]. Approximately 5 % of women have experienced RPL, and this proportion is even higher among women over 35 years of age [2]. Pregnancy loss has been described as a traumatic event for couples [3]. One-third of women with RPL are diagnosed as depressed [4, 5] and 21 % of them have levels of anxiety equal to or higher than that of a typical psychiatric outpatient [4], which in turn may affect sexual functioning and couples’ relationships[6]. Moreover, RPL women experiences higher depression and more impaired sexual function compared with the primigravidae [7].

RPL also impacts men; however, their grieving period is shorter than that of women [8]. In a survey of 40 men, 40–59 % reported a deepened awareness of the fragility of life, mourned the loss of their family’s hopes and dreams and experienced a strong sense of vulnerability and powerlessness [9], although the levels of distress among men are typically less intense than those of women [5, 10]. RPL also brings negative changes in self-concept and self-esteem [6]. Coping with involuntary childlessness and the stressors associated with treatment may contribute to the difficulties in a couple’s relationship and consequently lead to sexual dissatisfaction [11].

However, information about the impacts of RPL on men’s psychological status, sexual satisfaction and erectile function is scarce, and most of the previous studies have analyzed women’s perspectives exclusively. Hence, the aims of the present study are: (1) to describe the consequences of RPL on the psychological status, sexual satisfaction and erectile function of men; and (2) to explore factors that may contribute to the development of sexual dissatisfaction and psychological dysfunction.

## Methods

### Subjects

Subjects were recruited at two large, general, publicly owned hospitals: Shenzhen People’s Hospital and Peking University People's Hospital. Male patients whose partners experience RPL and an equal number of non-RPL male volunteers were recruited. All men had to meet the following criteria: (1) age ≥18 years, (2) in a stable heterosexual relationship for at least 1 year, and (3) able to read and speak Chinese. Their medical histories were evaluated carefully to rule out mental and/or other major medical diseases (e.g., thyroid dysfunction and diabetes mellitus). Men on medication that could have affected their ejaculatory and erectile function and/or psychological status were excluded (e.g., selective serotonin reuptake inhibitors, tricycle antidepressants and phosphodiesterase type 5 inhibitors). The study was approved by the ethical review board at Shenzhen People’s Hospital and Peking University People's Hospital. All subjects provided their written informed consent for participation in the study and for the data to be published in the future, and were guaranteed anonymity during data processing.

### Procedure

Subjects completed a psychological questionnaire and a demographic questionnaire including reproductive history. Forms were handed out upon their first visits to the clinic, prior to the investigation of the causes for RPL. Some subjects completed the questionnaires at the clinic; others took them home and returned them to the clinic by mail or E-mail.

### Measures

Personal data from the questionnaire included demographic variables such as age, marital status, height, weight, education level, monthly salary, and stress. To assess psychological burden of all men, the Chinese-language versions of two standardized and validated questionnaires for depression and anxiety [12] were administered: the Self-rating Anxiety Scale (SAS) [13] and the Self-rating Depression Scale (SDS) [14]. Each questionnaire contained 20 questions. We multiplied the total score on each questionnaire by 1.25. According to the Chinese norms [12], SAS ≥50 and SDS ≥53 represent diagnosable anxiety and depression, respectively. In addition, the severity of anxiety was categorized as [12]: mild (score 50 – 59), moderate (score 60 – 69), or severe (score ≥70). The category ranges for depression were: mild (53 – 62), moderate (63 – 72) or severe (≥72).

Sexual satisfaction was assessed with the Index of Sexual Satisfaction (ISS) [15], which evaluates respondents’ sexual satisfaction with their “real life” relationship. Measures of sexuality-related aspects for husbands address feelings, attitudes and events occurring in the relationship. The ISS comprises 25 items, all of which are statements describing the quality of the respondent's sex life (e.g., I feel that my partner enjoys our sex life). Respondents use seven-point Likert scales to rate the degree to which they agree with each item, with the end-points designated as “All the time” (Score 7) and “None of the time” (Score 1). The score is determined by summing all the item scores, subtracting the number of completed items, multiplying this figure by 100, and dividing the number of items completed by 6. This will produce a range from 0 to 100 with higher scores indicating a greater magnitude or severity of problems. Scores below or equal to 30 indicate an absence of a clinically significant problem. Scores above 30 indicate the likelihood of a clinically significant problem. Scores above 70 nearly always indicate that clients are experiencing severe stress with a clear possibility that some type of violence could be considered or used to deal with problems. In this study, the internal consistency of the Chinese-language ISS was 0.96.

Finally, on the basis of sexual activity status in the preceding four weeks, all men were required to complete the Chinese version [16] of the International Index of Erectile Function (IIEF-5) questionnaire [17]. Erectile dysfunction (ED) was divided into four severity grades [17]: no ED (IIEF-5score, 22 – 25), mild (12 – 21), moderate (8 –11), or severe (5 – 7).

### Statistical analyses

All statistical analyses were performed using SPSS software (SPSS Inc., Chicago, IL, USA) version 13.0. Descriptive statistics were used to summarize the demographic characteristics of the RPL and control groups. Numerical data, normally distributed after verified by the Kolmogorov-Smirnov test and expressed as mean ± SD, were compared by unpaired two-sided Student's t tests. The Chi-square test was used to compare categorical data. Correlations between the outcomes of the measures for sexual dysfunction and psychological burden in the RPL group and the control group were assessed using partial correlations. Logistic regression was applied for multivariate analysis. Odds ratios (OR) and 95 % confidence intervals (CI) were calculated to examine association strength. Because the ages of RPL men ranged from 20 to 49 years, and sexual dysfunctions are known to be age-dependent phenomena [18], all data were adjusted for age when the correlations between the sexual and psychological problems were assessed. For all of the tests, *P* < 0.05 was deemed statistically significant.

## Results

### Descriptive results

From January 2013 to December 2014, 260 men whose partners experience RPL were received the set of study questionnaires at their first clinic visit. A total of 236 men (response rate, 90.8 %) completed questionnaires. Another 236 male volunteers who had at least one child were enrolled from our health examination center as a control group. Men in RPL group discontinued the study for the following reasons: withdrawal of consent (*N* = 12), loss to follow-up (*N* = 8), incomplete information (*N* = 2), and other reasons (*N* = 2). The background characteristics of the participants are summarized in Table 1.

**Table 1:** Demographics information of men in RPL group and control group

Characteristics	RPL group	Control group	*P* value
	(*N* = 236)	(*N* = 236)	
Age, years			0.297
20-29	137 (58.1 %)	142 (60.2 %)	
30-39	63 (26.7 %)	61 (25.8 %)	
40-49	36 (15.3 %)	33 (14.0 %)	
BMI, kg/m2	25.32 ± 5.27	25.51 ± 5.01	0.802
Cigarette smoking			0.424
Yes	94 (39.8 %)	102 (43.2 %)	
No	142 (60.2 %)	134 (56.8 %)	
Alcohol consumption			
Yes	65 (27.5 %)	72 (30.5 %)	
No	171 (72.5 %)	164 (69.5 %)	
Educational status			0.794
Primary school	15 (6.4 %)	12 (5.0 %)	
Middle school	39 (16.5 %)	42 (17.8 %)	
High school	78 (33.1 %)	83 (35.2 %)	
University	104 (44.0 %)	99 (42.0 %)	
Monthly income (RMB)			0.507
< 3000	51 (21.6 %)	59 (25.0 %)	
3000-5000	79 (33.5 %)	81 (34.3 %)	
> 5000	106 (44.9 %)	96 (40.7 %)	
Length of marriage (year)	4.43 ± 3.77 (1–19)	4.81 ± 3.92 (1–21)	0.213
Number of past pregnancy losses	2.90 ± 0.97 (2–6)	N/A	
Interval after the latest loss		N/A	
< 1 month	46 (19.49 %)		
1-3 months	122 (51.69 %)		
4-6 months	57 (24.15 %)		
> 6 months	11 (4.66 %)		

Data were expressed as mean ± standard deviation or number (percentage) or range, when appropriate

Data were assessed with one-way ANOVA or Chi-square test, when appropriate

*BMI* body mass index, 1 US dollar = 6.5 RMB

When compared with men from the control group, men in RPL group reported significantly higher rates of sexual dysfunction and psychological burden, including ED, anxiety, and depression symptoms (*P* < 0.001 for all) (Table 2).

**Table 2:** IIEF-5, SAS, and SDS scores of men in RPL group and control group

Characteristics	RPL group	Control group	*P* value
ISS	58.01 ± 15.11	39.32 ± 12.27	0.021
IIEF-5, scores			<0.001
5-7	0 (0 %)	0 (0 %)	
8-11	16 (6.78 %)	3 (1.27 %)	
12-21	29 (12.29 %)	15 (6.36 %)	
22-25	191 (80.93 %)	218 (92.37 %)	
Mean ± SD	21.46 ± 5.96	24.61 ± 4.71	0.010
SAS, scores			<0.001
< 50	149 (63.14 %)	79.24 (21.6 %)	
50-59	48 (20.33 %)	25 (10.59 %)	
60-69	22 (9.32 %)	16 (6.78 %)	
≥ 70	17 (7.20 %)	8 (3.39 %)	
Mean ± SD	39.11 ± 15.73	24.23 ± 16.37	<0.001
SDS, scores			<0.001
<53	174 (73.73 %)	218 (92.37 %)	
53-62	25 (10.59 %)	13 (5.51 %)	
63-72	37 (15.68 %)	5 (2.12 %)	
≥72	0 (0 %)	0 (0 %)	
Mean ± SD	26.32 ± 17.66	12.79 ± 8.30	<0.001

Data were expressed as mean ± standard deviation or number (percentage), when appropriate

Difference between RPL and control groups assessed by Chi-square test or t-test, as appropriate

*ISS* index of sexual satisfaction, *IIEF-5* international index of erectile function, *SAS* self-rating anxiety scale, *SDS* self-rating depression scale

The mean ISS scores for the RPL group were higher than those for the control group (*P* = 0.021), indicating that the men from the RPL group were having poorer sexual contact experiences. In this group, 19 (8.05 %) men had scores over 70, which indicated they may have been having significant stress in their daily life.

When erectile function was assessed, the mean IEFF-5 scores for the RPL were lower than the control groups (*P* = 0.010). In the RPL group, a total of 45 men (19.07 %) reported overall ED (IIEF-5 score <22), and twenty-nine men reported a mild form (IIEF-5 score 12 – 21) and 16 a moderate form (score 8 – 11), whereas no patients reported a severe form (score 5 – 7). However, in the control group, 18 men (7.63 %) were diagnosed with ED. Fifteen (6.36 %) men reported mild ED and three (1.27 %) a moderate ED, whereas no men reported a severe ED.

According to the SAS and SDS results, 36.90 % (87/236) and 26.30 % (62/236) men of RPL group were diagnosed with anxiety and depression, respectively. Mean scores of the SAS and SDS for men with RPL partners were presented in Table 2. Among them, 20.33 % (48/236) reported mild anxiety, 9.32 % (22/236) moderate anxiety, and 7.20 % (17/236) severe anxiety. For the SDS, 10.60 % (25/236) reported mild depression and 15.68 % (37/236) moderate depression, whereas no patients reported severe depression. Additionally, the SAS and SDS scores were not reliably associated with the number of past pregnancy losses.

In contrast, the mean SAS and SDS scores reported by men in the control group were lower than those in the RPL group (*P* < 0.001). The incidences of anxiety and depression in the control group were 19.08 % (49/236) and 7.63 % (18/236), respectively, which were lower than those in the RPL group (*P* < 0.001 for all).

In addition, there were several associations among the sexual function and psychological distress variables (Table 3). After adjusting for age, negative relationships were observed between the IIEF-5 score and both SAS and SDS scores; positive relationships were observed between the ISS score and both SAS and SDS scores. Furthermore, plausible predictors for ED (IIEF-5 < 22) were examined using a multivariate logistic regression model. We found that age, whose partners experience a history of RPL, anxiety and depressive symptoms were significantly associated with a higher risk of ED (Table 4).

**Table 3:** Associations between the outcomes of the measures for sexual function and psychological distress

Characteristics	IIEF-5		ISS	
	Adjusted r	*P*	Adjusted r	*P*
SAS	-0.40	<0.001	0.49	<0.001
SDS	-0.52	<0.001	0.50	<0.001

**Table 4:** Multivariate logistic regression analysis predicting factors associated with ED (IIEF-5<22)

Covariates	OR	95 % CI	*P* value
Age	1.10	1.03-1.24	<0.001
Cigarette smoking	1.20	0.93-1.58	0.142
Alcohol consumption	0.96	0.90-1.06	0.258
RPL history	1.47	1.18-2.29	<0.001
Anxiety	1.86	1.52-2.78	<0.001
Depression	1.58	1.28-2.64	<0.001

## Discussion

This study focused on the consequences of RPL on psychological status, sexual satisfaction, and erectile function, and their possible interrelations among men in China. Results from our study showed that the incidences of ED and psychological problems (anxiety and depression) in men whose partners experience RPL were higher than those of men in a control group, and a significant association was found between sexual and psychological problems in men with RPL partners.

Our study found that men with RPL partners reported higher incidences of ED and lower sexual satisfaction than did the control group. These incidences were also higher than those reported in a comparable study [19] on the Chinese general population (ED: 13 %) with subjects of a similar mean age. Similarly, several studies conducted of infertile male patients have reported very similar outcomes. Men in couples who fail to have children experience higher levels of anxiety and depressive symptoms than do fertile men [20]. Similar results have also been published by Dyer et al. [21] from South Africa, where male partners in infertile couples experienced significantly elevated levels of psychological distress compared with fertile men. In addition, ED is reported by one in six infertile patients, mainly associated with depressive symptoms [22]. These findings confirm the data from other studies [21, 23], which suggest that the incidences of sexual and psychological problems in infertile men are higher than those in fertile men. However, it should be noted that different diagnostic criteria for sexual and psychological disorders exist among the various professional organizations and/or individuals. Moreover, the cultural and religious differences between Chinese and Western patient populations should be taken into account. Hence, the difference in prevalence rates can be explained by the previously mentioned factors, and further research is needed to confirm and extend these results.

Although, mechanisms between sexual and psychological problems have not been well studied. The etiology of ED is a combination of psychosocial and organic factors. Corona et al. [24] found that negative psychological factors (e.g., loss or reduced job satisfaction) might impair self-esteem, pushing men to feel disgraced, weakened, and frightened, all of which are negative feelings that can extend further into sexual life, bringing impairments there as well and causing further frustration. Recently, they developed and validated a New SIEDY Scale 3, which is a rapid case history tool able to detect the psychopathological symptoms in patients suffering from ED, allowing the identification of those subjects who should undergo an in-depth psychiatric evaluation [25]. Accordingly, we speculate that with the recurrent loss of pregnancies, infertile men might develop feelings of guilt, anxiety and depression. These negative psychological factors might further affect their sexual functioning. Furthermore, diagnostic procedures and treatment attempts may increase psychological distress and hasten the decline in sexual satisfaction. Sexual dissatisfaction could also be related to a withdrawal from sexual activities and hence to even lower chances of conception. However, the cross-sectional nature of our study could not clarify the causal relationship between sexual dysfunction and psychological burden, so further studies on their mechanisms are needed.

## Conclusions 

This study was limited by inclusion of a small number of subjects, being a single facility cross-sectional study, and by the use of only self-report assessments. We do not know whether our results can be extended to individuals seeking treatment at other clinics or to those who do not seek treatment at all. Therefore, further in-depth studies on large epidemiological populations are needed to confirm and extend these results. Despite these limitations, this study is noteworthy because, to the best of our knowledge, it is the first research that has comprehensively assessed psychological burden, sexual satisfaction and erectile dysfunction among men whose partners experienced RPL. These findings collectively suggest the importance of psychological care following RPL. Evaluation and management of psychological and psychosocial adjustment of men, and not just women, should be considered in the treatment of RPL to enable partners to adjust emotionally to past pregnancy losses as well as to future pregnancies.
